# Prime editing enables precise genome modification of a *Populus* hybrid

**DOI:** 10.1007/s42994-024-00177-1

**Published:** 2024-09-06

**Authors:** Jinpeng Zou, Yuhong Li, Kejian Wang, Chun Wang, Renying Zhuo

**Affiliations:** 1 State Key Laboratory of Tree Genetics and Breeding, Key Laboratory of Tree Breeding of Zhejiang Province, Research Institute of Subtropical Forestry, Chinese Academy of Forestry, Hangzhou, 311400 China; 2 State Key Laboratory of Rice Biology and Breeding, China National Rice Research Institute, Chinese Academy of Agricultural Sciences, Hangzhou, 310006 China; 3 Key Laboratory of Gene Editing Technologies (Hainan), Ministry of Agriculture and Rural Affairs, Sanya, 572025 China

**Keywords:** Poplar, Prime editing, PE3 system, Dicot

## Abstract

CRISPR/Cas-based genome editing has been extensively employed in the breeding and genetic improvement of trees, yet precise editing remains challenging in these species. Prime editing (PE), a revolutionary technology for precise editing, allows for arbitrary base substitutions and the insertion/deletion of small fragments. In this study, we focused on the model tree poplar 84K (*Populus alba* × *P. glandulosa*). We used the *2* × *35S* promoter to express a fusion protein of spCas9 nickase (nCas9) and engineered Moloney murine leukemia virus (MMLV), and the *Arabidopsis thaliana AtU6* promoter to express an engineered PE guide RNA (epegRNA) and Nick gRNA, pioneering the establishment of the Prime Editor 3 (PE3) system in dicot poplar. Single-base substitutions, multiple-base substitutions, and small-fragment insertions/deletions were edited into three endogenous target genes. The desired edits were identified in hygromycin-resistant (transformed) calli at seven out of nine target sites, with an average editing efficiency ranging from 0.1 to 3.6%. Furthermore, stable T_0_ plants contained the desired edits at four out of nine targets, with editing efficiencies ranging from 3.6 to 22.2%. Establishment of the PE3 system provides a powerful tool for the precise modification of the poplar genome.

**Supplementary Information:**

The online version contains supplementary material available at 10.1007/s42994-024-00177-1.

Dear Editor,

Trees are an invaluable resource on Earth, providing substantial economic and ecological benefits. CRISPR/Cas-based genome editing has tremendous potential for the breeding and genetic improvement of tree species (Sulis et al. 2023; Zhang et al. 2023b). Poplar (*Populus*) species are excellent dicot tree models and have been extensively studied; however, precise genome editing of these plants remains challenging. Recently, a revolutionary precise editing technology called “prime editing (PE)” has emerged, enabling user-defined DNA sequence alterations including arbitrary base substitutions and small-fragment insertions/deletions (Anzalone et al. 2019). Known for its accuracy and versatility, PE has been extensively applied in mammalian cells and major crops (Anzalone et al. 2019; Jiang et al. 2020; Li et al. 2022a; Lin et al. 2020; Lu et al. 2021); however, the long generation cycles, high genomic heterozygosity, and poor genetic transformation stability of trees have hampered its use in these plants. Here, we successfully applied PE to dicot poplar using a Prime Editor 3 (PE3) system (Fig. 1A).

**Fig. 1:**
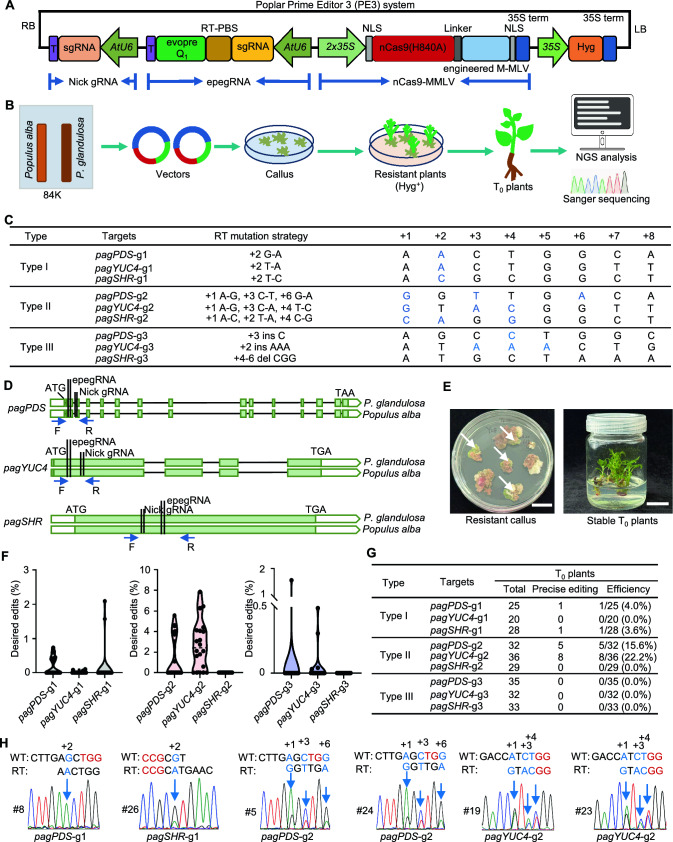
Prime editing in poplar. **A** Schematic diagram of the poplar PE3 system. It comprises three components: nCas9-MMLV, epegRNA, and Nick gRNA. **B** Plant genetic transformation process and evaluation of editing efficiency in poplar 84K. **C** Sequence after PE using RT templates with mutated bases, counting the first base 3′ of the epegRNA-induced nick as position + 1. Base mutations are highlighted in blue. Type I: single-base mutation. Type II: multiple-base mutation. Type III: small-fragment insertion/deletion. **D** Location of epegRNA and Nick gRNA. The two gene models represent the genomes of *P. alba* and *P. glandulosa*. Blue arrows indicate the location of specific primers. **E** Phenotypes of resistant calli and stable transgenic T_0_ plants. Bars = 2 cm. **F** Efficiency of precise editing at nine targets in stably transformed calli. Efficiencies were calculated from the ratios of edited reads to total clean reads via a Hi-TOM analysis (*n* = 20). **G** Frequencies of successful PE in T_0_ plants using the PE3 system. Frequencies = (number of plants with the desired edit)/(number of total plants). **H** Sanger sequencing of representative desired edits in T_0_ plants. Red letters represent the PAM sequence, blue letters represent mutated bases, and numbers and arrows indicate the positions of mutations

The poplar PE3 system was based on our previously established efficient PPE3-evopreQ_1_ system in monocot rice (*Oryza sativa*) (Zou et al. 2022) and the CRISPR/Cas system in poplar (An et al. 2020, 2021). It comprises three components: nCas9-MMLV (a fusion protein of spCas9 nickase and engineered Moloney murine leukemia virus), epegRNA (an engineered PE guide RNA containing single-guide RNA (sgRNA), a primer binding site (PBS), and a reverse transcriptase (RT) template with editing and RNA motif evopreQ_1_), and Nick gRNA (a sgRNA for DNA nicking) (Fig. 1A, Fig. S1). The *Arabidopsis thaliana AtU6* promoter was used to efficiently express the epegRNA and Nick gRNA, while the nCas9-MMLV was expressed under the control of the *2* × *35S* promoter. Subsequently, the PE3 system was transformed into a poplar hybrid (*Populus alba* × *P. glandulosa* hybrid clone poplar 84K) via *Agrobacterium*-mediated transformation, and PE efficiencies were estimated (Fig. 1B).

Three endogenous genes were selected as PE targets: *PHYTOENE DESATURASE* (*PagPDS*; *Potri.014G148700*), a homolog of *Arabidopsis AtYUCCA4* (*PagYUC4*; *Potri.006G248200*), and *SHORT ROOT* (*PagSHR*; *Potri.007G063300*) (An et al. 2020; Triozzi et al. 2021). To assess the editing capabilities of this method, we designed three types of RT template: Type I contained only one single-base substitution, Type II involved multiple-base substitutions, and Type III consisted of small DNA fragment insertions/deletions (Fig. 1C; Fig. S2; Table S1). Poplar 84K is a hybrid diploid with allelic variations; thus, all epegRNAs and Nick gRNAs were designed to target both the *P. alba* and *P. glandulosa* genomes to avoid editing failure resulting from mismatches between the target sites and genomic sequences (Fig. 1D).

To assess the feasibility of the PE3 system in 84K early on, we prioritized evaluating PE efficiencies in resistant callus. Twenty independent resistant calli were selected as individual replicates for each target and subjected to separate DNA extraction and next-generation sequencing (NGS) analysis using a Hi-TOM platform (Fig. 1E, Table S2) (Sun et al. 2024). We calculated the proportion of successful precise editing in each individual callus, and averaged the editing efficiency across all edited calli to determine the editing efficiency at that target site (Fig. 1F). For Type I mutations, all three targets exhibited the desired edits with average efficiencies of 0.49% (*PagPDS*), 0.08% (*PagYUC4*), and 1.83% (*PagSHR*). For Type II edits, the *PagPDS*-g2 and *PagYUC4*-g2 targets were edited with average efficiencies of 3.82% and 3.62%, respectively, but the *PagSHR*-g2 target did not exhibit any desired edits. Desired Type III edits were detected for the *PagPDS*-g3 and *PagYUC4*-g3 targets, with average efficiencies of 1.59% and 0.22%, respectively (Fig. 1F). These results demonstrated that the PE3 system is capable of completing PE; however, as only a small number of cells are edited in one callus, the efficiencies achieved do not represent the editing efficiency in T_0_ plants. Relatively low efficiencies of Type III edits compared with those of Type I and Type II are consistent with previous findings in monocot rice and wheat (*Triticum aestivum*) (Lin et al. 2020).

To validate the reliability of PE, we regenerated stable transgenic T_0_ plants for all targets (Fig. 1E) and used NGS to identify the editing types in all plants. The desired edits were detected at the *PagPDS*-g1 and *PagSHR*-g1 Type I targets, as well as at the *PagPDS*-g2 and *PagYUC4*-g2 Type II targets (Fig. 1G). T_0_ plants with the desired edits were selected for further confirmation using Sanger sequencing (Fig. 1H; Fig. S3, Table S3). We identified one desired edit (1/25, 4.0%; homozygous) at the *PagPDS*-g1 target and five desired edits (5/32, 15.6%; chimeric) at the *PagPDS*-g2 target. No desired edits (0/35, 0.0%) were identified at the *PagPDS*-g3 target. Desired edits at the *PagPDS* sites were limited to base substitutions of Type I and Type II, resulting in no observed plant albinism. For *PagYUC4* and *PagSHR* targets, desired edits were obtained at the Type I *PagSHR*-g1 target (1/28, 3.6%; heterozygous) and the Type II *PagYUC4*-g2 target (8/36, 22.2%; two heterozygous and six chimeric) (Fig. 1G, 1H; Fig. S3). This indicates that resistant calli with high PE efficiency are likely to differentiate into edited T_0_ plants, representing an even higher editing efficiency, consistent with previous studies in rice (Zou et al. 2022). Type I and Type II edits were obtained with greater efficiency than Type III mutations, consistent with the results in resistant callus (Fig. 1F). Beyond the desired edits, only one byproduct was detected at the *PagPDS-*g1 target, with no byproducts detected at the other targets (Fig. S4).

In summary, we successfully established a PE3 system in dicot poplar and obtained stable T_0_ plants with the desired edits. Currently, the efficiency of the PE3 system in poplar is low and unstable, particularly for small-fragment insertions/deletions. Moreover, PE-mediated precise editing still leads to a high proportion of chimerism (Fig. 1G, 1H), which significantly hinders the effective application and advancement of PE technology in poplar. These issues could be attributed to the leaf disk method used for genetic transformation, which results in poor regenerative capacity of the callus and consequently impacts the performance of PE. Furthermore, the components of the PE system, apart from the promoter, are sourced from monocot rice and may not be optimal for dicot poplar. To date, the PE system has only been established in three dicot plants, tomato (*Solanum lycopersicum*), potato (*Solanum tuberosum*), and tobacco (*Nicotiana tabacum*), for which efficiencies are also low (Lu et al. 2021; Perroud et al. 2022; Zhang et al. 2023a). PE systems for dicot plants thus require further improvement if they are to be broadly used for basic research and precise breeding. The PE3 system could be further optimized through methods effective in major crops, such as increasing epegRNA expression, enhancing MMLV activity, or implementing appropriate heat treatments (Jiang et al. 2020; Li et al. 2022b; Zong et al. 2022; Zou et al. 2022).

## Materials and methods

### Vector construction

The poplar PE3 system comprised three vectors: pC1300-PE, SK-epegRNA, and SK-Nick gRNA. In the pC1300-PE binary vector, the *2* × *35S* promoter was used to express the nCas9-MMLV fusion protein. The *AtU6-26* promoter was used to express the epegRNA in the SK-epegRNA vector and the Nick gRNA in the SK-Nick gRNA vector. The Nick gRNA fragment (digested with *Xho*I and *Bgl*II) and the epegRNA fragment (digested with *Kpn*I and *Sal*I) were assembled into the pC1300-PE vector (digested with *Kpn*I and *Bam*HI) using T4 ligase to obtain the PE3 system. Sequences of the three vectors are shown in Fig. S1.

### Design of pegRNAs

*PagPDS*, *PagYUC4*, and *PagSHR* were identified from the *Populus trichocarpa* genome in the Phytozome v13 database (https://phytozome-next.jgi.doe.gov/). Specific primers were designed to amplify the target sequences (Table S2; Table S3). Three types of pegRNAs were randomly designed for each target sequence: single-base substitutions, multiple-base substitutions, and small-fragment insertions/deletions. The pegRNA design scheme was optimized using the PlantPegDesigner website, which was developed specifically for plants (Jin et al. 2023).

### *Agrobacterium*-mediated callus transformation of poplar 84K

*Agrobacterium tumefaciens* strain EHA105 harboring the binary expression vector PE3 containing the hygromycin (Hyg^+^) reporter gene was used for genetic transformation of poplar 84K calli, which was performed as previously reported with some modifications (Wen et al. 2022). Rapidly growing and well-separated calli were used for transformation. After infection, calli were selected using 2.5 mg/L hygromycin B for 5 weeks to obtain resistant calli, which were further differentiated into stable transgenic T_0_ plants. One T_0_ plant from each transgenic event was selected for rooting and genotyping. Plant materials were grown under a 16:8-h light:dark photoperiod at 25 °C.

### Sampling and genotyping

Genomic DNA of resistant calli and T_0_ plants was extracted using the cetyltrimethyl ammonium bromide (CTAB) method and subjected to NGS analysis using the Hi-TOM platform (Sun et al. 2024). PE efficiency in resistant calli = (number of reads with the desired edits)/(number of total reads). Frequency of PE in transgenic T_0_ plants = (number of plants with the desired edits)/(number of total plants). Mutation reads representing less than 5% of reads in transgenic T_0_ plants were filtered out during data analysis. The desired mutations in T_0_ plants were validated using PCR and Sanger sequencing. A mutation frequency ≥ 70% was considered a homozygous mutation, ≥ 30% and < 70% was a heterozygous mutation, ≥ 5% and < 30% was a chimeric mutation, and < 5% was counted as the wild type. Primers used in this study are listed in Table S2 and Table S3.

### Statistical analysis

All data were analyzed using GraphPad Prism 8.0.2 software.

## Supplementary Information

Below is the link to the electronic supplementary material.

Supplementary file1 (DOCX 510 KB)

## Data Availability

The authors confirm that all data from this study are available and can be found in this article and in the supplementary information.
