# Supplementary material for: Prime editing enables precise genome modification of a Populus hybrid
Source: aBIOTECH. 2024 Sep 6;5(4):497–501. doi: 10.1007/s42994-024-00177-1 (PMC11624180; doi:10.1007/s42994-024-00177-1)
Supplement: Supplementary file 1 — Supplementary file1 (DOCX 510 KB) [file 42994_2024_177_MOESM1_ESM.docx]

**Prime editing enables precise genome modification of a *Populus* hybrid**

Jinpeng Zou^1,2,†^, Yuhong Li^1,†^, Kejian Wang^2,3^, Chun Wang^2,*^, Renying Zhuo^1,*^

^1^State Key Laboratory of Tree Genetics and Breeding, Key Laboratory of Tree Breeding of Zhejiang Province, Research Institute of Subtropical Forestry, Chinese Academy of Forestry, Hangzhou 311400, China

^2^State Key Laboratory of Rice Biology and Breeding, China National Rice Research Institute, Chinese Academy of Agricultural Sciences, Hangzhou 310006, China

^3^Key Laboratory of Gene Editing Technologies (Hainan), Ministry of Agriculture and Rural Affairs, Sanya 572025, China

^†^Jinpeng Zou and Yuhong Li have contributed equally to this work.

^*^Correspondence: [zhuory@gmail.com](mailto:zhuory@gmail.com) (R. Zhuo) and [wangchun@caas.cn](mailto:wangchun@caas.cn) (C. Wang).

**Supporting information**

**Fig. S1** Complete sequences of the pC1300-PE, SK-epegRNA and SK-Nick gRNA vectors.

**Fig. S2** Schematic illustrations of pegRNAs design.

**Fig.S3** Sanger sequencing of other representative desired edits in T_0_ plants.

**Fig. S4** Alignments of PE byproducts at *pagPDS-g1* site in T_0_ plants.

Table 1 The pegRNA target sites, Nick sgRNA sites, RT template and PBS of different target sites.

Table 2 NGS primers were used to identify the precise editing types of the T_0_ plants.

Table 3 PCR primers for amplifying the target sites and Sanger sequence of T_0_ plants.

Supplemental Figure

A

pC1300-PE vector:

*Kpn*I *Bam*HI

GAGCTCGGTACCAAGGATCCGTACCCCTACTCCAAAAATGTCAAAGATACAGTCTCAGAAGACCAAAGGGCTATTGAGACTTTTCAACAAAGGGTAATTTCGGGAAACCTCCTCGGATTCCATTGCCCAGCTATCTGTCACTTCATCGAAAGGACAGTAGAAAAGGAAGGTGGCTCCTACAAATGCCATCATTGCGATAAAGGAAAGGCTATCATTCAAGATGCCTCTGCCGACAGTGGTCCCAAAGATGGACCCCCACCCACGAGGAGCATCGTGGAAAAAGAAGACGTTCCAACCACGTCTTCAAAGCAAGTGGATTGATGTGACATCTCCACTGACGTAAGGGATGACGCACAATCCCACCCCTACTCCAAAAATGTCAAAGATACAGTCTCAGAAGACCAAAGGGCTATTGAGACTTTTCAACAAAGGGTAATTTCGGGAAACCTCCTCGGATTCCATTGCCCAGCTATCTGTCACTTCATCGAAAGGACAGTAGAAAAGGAAGGTGGCTCCTACAAATGCCATCATTGCGATAAAGGAAAGGCTATCATTCAAGATGCCTCTGCCGACAGTGGTCCCAAAGATGGACCCCCACCCACGAGGAGCATCGTGGAAAAAGAAGACGTTCCAACCACGTCTTCAAAGCAAGTGGATTGATGTGACATCTCCACTGACGTAAGGGATGACGCACAATCCCACTATCCTTCGCAAGACCCTTCCTCTATATAAGGAAGTTCATTTCATTTGGAGAGGACAGCCCAAGCTAGATCCATGGCCCCTAAGAAGAAGAGAAAGGTCGGTATTCACGGCGTTCCTGCGGCGATGGACAAGAAGTATAGTATTGGTCTGGACATTGGGACGAATTCCGTTGGCTGGGCCGTGATCACCGATGAGTACAAGGTCCCTTCCAAGAAGTTTAAGGTTCTGGGGAACACCGATCGGCACAGCATCAAGAAGAATCTCATTGGAGCCCTCCTGTTCGACTCAGGCGAGACCGCCGAAGCAACAAGGCTCAAGAGAACCGCAAGGAGACGGTATACAAGAAGGAAGAATAGGATCTGCTACCTGCAGGAGATTTTCAGCAACGAAATGGCGAAGGTGGACGATTCGTTCTTTCATAGATTGGAGGAGAGTTTCCTCGTCGAGGAAGATAAGAAGCACGAGAGGCATCCTATCTTTGGCAACATTGTCGACGAGGTTGCCTATCACGAAAAGTACCCCACAATCTATCATCTGCGGAAGAAGCTTGTGGACTCGACTGATAAGGCGGACCTTAGATTGATCTACCTCGCTCTGGCACACATGATTAAGTTCAGGGGCCATTTTCTGATCGAGGGGGATCTTAACCCGGACAATAGCGATGTGGACAAGTTGTTCATCCAGCTCGTCCAAACCTACAATCAGCTCTTTGAGGAAAACCCAATTAATGCTTCAGGCGTCGACGCCAAGGCGATCCTGTCTGCACGCCTTTCAAAGTCTCGCCGGCTTGAGAACTTGATCGCTCAACTCCCGGGCGAAAAGAAGAACGGCTTGTTCGGGAATCTCATTGCACTTTCGTTGGGGCTCACACCAAACTTCAAGAGTAATTTTGATCTCGCTGAGGACGCAAAGCTGCAGCTTTCCAAGGACACTTATGACGATGACCTGGATAACCTTTTGGCCCAAATCGGCGATCAGTACGCGGACTTGTTCCTCGCCGCGAAGAATTTGTCGGACGCGATCCTCCTGAGTGATATTCTCCGCGTGAACACCGAGATTACAAAGGCCCCGCTCTCGGCGAGTATGATCAAGCGCTATGACGAGCACCATCAGGATCTGACCCTTTTGAAGGCTTTGGTCCGGCAGCAACTCCCAGAGAAGTACAAGGAAATCTTCTTTGATCAATCCAAGAACGGCTACGCTGGTTATATTGACGGCGGGGCATCGCAGGAGGAATTCTACAAGTTTATCAAGCCAATTCTGGAGAAGATGGATGGCACAGAGGAACTCCTGGTGAAGCTCAATAGGGAGGACCTTTTGCGGAAGCAAAGAACTTTCGATAACGGCAGCATCCCTCACCAGATTCATCTCGGGGAGCTGCACGCCATCCTGAGAAGGCAGGAAGACTTCTACCCCTTTCTTAAGGATAACCGGGAGAAGATCGAAAAGATTCTGACGTTCAGAATTCCGTACTATGTCGGACCACTCGCCCGGGGTAATTCCAGATTTGCGTGGATGACCAGAAAGAGCGAGGAAACCATCACACCTTGGAACTTCGAGGAAGTGGTCGATAAGGGCGCTTCCGCACAGAGCTTCATTGAGCGCATGACAAATTTTGACAAGAACCTGCCTAATGAGAAGGTCCTTCCCAAGCATTCCCTCCTGTACGAGTATTTCACTGTTTATAACGAACTCACGAAGGTGAAGTATGTGACCGAGGGAATGCGCAAGCCCGCCTTCCTGAGCGGCGAGCAAAAGAAGGCGATCGTGGACCTTTTGTTTAAGACCAATCGGAAGGTCACAGTTAAGCAGCTCAAGGAGGACTACTTCAAGAAGATTGAATGCTTCGATTCCGTTGAGATCAGCGGCGTGGAAGACAGGTTTAACGCGTCACTGGGGACTTACCACGATCTCCTGAAGATCATTAAGGATAAGGACTTCTTGGACAACGAGGAAAATGAGGATATCCTCGAAGACATTGTCCTGACTCTTACGTTGTTTGAGGATAGGGAAATGATCGAGGAACGCTTGAAGACGTATGCCCATCTCTTCGATGACAAGGTTATGAAGCAGCTCAAGAGAAGAAGATACACCGGATGGGGAAGGCTGTCCCGCAAGCTTATCAATGGCATTAGAGACAAGCAATCAGGGAAGACAATCCTTGACTTTTTGAAGTCTGATGGCTTCGCGAACAGGAATTTTATGCAGCTGATTCACGATGACTCACTTACTTTCAAGGAGGATATCCAGAAGGCTCAAGTGTCGGGACAAGGTGACAGTCTGCACGAGCATATCGCCAACCTTGCGGGATCTCCTGCAATCAAGAAGGGTATTCTGCAGACAGTCAAGGTTGTGGATGAGCTTGTGAAGGTCATGGGACGGCATAAGCCCGAGAACATCGTTATTGAGATGGCCAGAGAAAATCAGACCACACAAAAGGGTCAGAAGAACTCGAGGGAGCGCATGAAGCGCATCGAGGAAGGCATTAAGGAGCTGGGGAGTCAGATCCTTAAGGAGCACCCGGTGGAAAACACGCAGTTGCAAAATGAGAAGCTCTATCTGTACTATCTGCAAAATGGCAGGGATATGTATGTGGACCAGGAGTTGGATATTAACCGCCTCTCGGATTACGACGTCGATGCTATCGTTCCTCAGTCCTTCCTTAAGGATGACAGCATTGACAATAAGGTTCTCACCAGGTCCGACAAGAACCGCGGGAAGTCCGATAATGTGCCCAGCGAGGAAGTCGTTAAGAAGATGAAGAACTACTGGAGGCAACTTTTGAATGCCAAGTTGATCACACAGAGGAAGTTTGATAACCTCACTAAGGCCGAGCGCGGAGGTCTCAGCGAACTGGACAAGGCGGGCTTCATTAAGCGGCAACTGGTTGAGACTAGACAGATCACGAAGCACGTGGCGCAGATTCTCGATTCACGCATGAACACGAAGTACGATGAGAATGACAAGCTGATCCGGGAAGTGAAGGTCATCACCTTGAAGTCAAAGCTCGTTTCTGACTTCAGGAAGGATTTCCAATTTTATAAGGTGCGCGAGATCAACAATTATCACCATGCTCATGACGCATACCTCAACGCTGTGGTCGGAACAGCATTGATTAAGAAGTACCCGAAGCTCGAGTCCGAATTCGTGTACGGTGACTATAAGGTTTACGATGTGCGCAAGATGATCGCCAAGTCAGAGCAGGAAATTGGCAAGGCCACTGCGAAGTATTTCTTTTACTCTAACATTATGAATTTCTTTAAGACTGAGATCACGCTGGCTAATGGCGAAATCCGGAAGAGACCACTTATTGAGACCAACGGCGAGACAGGGGAAATCGTGTGGGACAAGGGGAGGGATTTCGCCACAGTCCGCAAGGTTCTCTCTATGCCTCAAGTGAATATTGTCAAGAAGACTGAAGTCCAGACGGGCGGGTTCTCAAAGGAATCTATTCTGCCCAAGCGGAACTCGGATAAGCTTATCGCCAGAAAGAAGGACTGGGACCCGAAGAAGTATGGAGGTTTCGACTCACCAACGGTGGCTTACTCTGTCCTGGTTGTGGCAAAGGTGGAGAAGGGAAAGTCAAAGAAGCTCAAGTCTGTCAAGGAGCTCCTGGGTATCACCATTATGGAGAGGTCCAGCTTCGAAAAGAATCCGATCGATTTTCTCGAGGCGAAGGGATATAAGGAAGTGAAGAAGGACCTGATCATTAAGCTTCCAAAGTACAGTCTTTTCGAGTTGGAAAACGGCAGGAAGCGCATGTTGGCTTCCGCAGGAGAGCTCCAGAAGGGTAACGAGCTTGCTTTGCCGTCCAAGTATGTGAACTTCCTCTATCTGGCATCCCACTACGAGAAGCTCAAGGGCAGCCCAGAGGATAACGAACAGAAGCAACTGTTTGTGGAGCAACACAAGCATTATCTTGACGAGATCATTGAACAGATTTCGGAGTTCAGTAAGCGCGTCATCCTCGCCGACGCGAATTTGGATAAGGTTCTCTCAGCCTACAACAAGCACCGGGACAAGCCTATCAGAGAGCAGGCGGAAAATATCATTCATCTCTTCACCCTGACAAACCTTGGGGCTCCCGCTGCATTCAAGTATTTTGACACTACGATTGATCGGAAGAGATACACTTCTACGAAGGAGGTGCTGGATGCAACCCTTATCCACCAATCGATTACTGGCCTCTACGAGACGCGGATCGACTTGAGTCAGCTCGGGGGGGATAAGAGACCAGCGGCAACCAAGAAGGCAGGACAAGCGAAGAAGAAGAAGTCTGGCGGCTCCTCTGGTGGATCTTCTGGTTCTGAAACTCCAGGTACGAGCGAAAGCGCCACGCCCGAAAGCAGCGGTGGTTCGTCTGGCGGATCTTCCACTCTCAATATCGAAGACGAGTACAGGCTGCACGAGACGTCAAAGGAACCTGACGTCAGCCTGGGTTCCACTTGGTTGTCGGATTTCCCGCAGGCATGGGCTGAAACCGGCGGCATGGGCTTGGCCGTGCGGCAGGCTCCTCTGATAATCCCGCTGAAGGCCACATCCACCCCGGTGTCGATTAAGCAATACCCTATGTCCCAAGAAGCTCGGTTGGGTATCAAGCCGCACATTCAGCGGCTGTTGGACCAGGGCATCCTTGTTCCCTGTCAGTCGCCGTGGAATACTCCGCTGTTGCCCGTGAAGAAGCCGGGCACAAATGATTATCGGCCCGTCCAAGATCTTCGCGAGGTGAACAAGAGAGTGGAGGATATTCATCCGACCGTCCCTAATCCTTATAATCTTCTGTCAGGCTTGCCACCCTCGCACCAGTGGTACACAGTGTTGGATCTTAAGGACGCGTTCTTCTGCCTCCGGCTTCATCCAACGTCGCAGCCTCTGTTCGCGTTTGAATGGAGGGACCCTGAGATGGGCATATCCGGACAGCTGACATGGACCCGCCTCCCTCAAGGCTTCAAGAACTCGCCGACGCTCTTTAACGAGGCTCTCCACAGAGATCTCGCTGACTTTAGGATTCAACACCCAGACCTCATTCTTCTGCAGTATGTCGATGACCTCCTGCTTGCAGCTACATCTGAGCTCGATTGCCAACAAGGAACTAGAGCTTTGCTGCAAACCCTTGGGAATCTTGGATATCGGGCATCGGCAAAGAAAGCACAAATTTGTCAAAAGCAAGTCAAATACTTGGGCTACCTCCTTAAGGAGGGACAACGCTGGCTCACTGAAGCCCGCAAAGAGACAGTGATGGGACAGCCGACGCCAAAGACGCCTCGCCAGTTGAGGGAGTTCCTGGGCAAAGCCGGTTTCTGCAGGCTCTTTATTCCGGGTTTCGCGGAAATGGCAGCTCCTTTGTACCCGCTCACGAAGCCGGGCACGCTGTTCAACTGGGGGCCTGACCAGCAGAAGGCTTACCAAGAAATTAAACAGGCTCTTCTTACGGCTCCGGCTCTTGGGCTGCCTGACCTTACTAAACCGTTTGAGCTCTTCGTTGACGAGAAGCAAGGATACGCAAAGGGCGTCCTCACTCAGAAGCTTGGGCCTTGGAGACGGCCCGTTGCATACCTGAGCAAGAAGCTCGACCCAGTGGCGGCGGGTTGGCCACCTTGTCTTCGGATGGTGGCTGCTATTGCGGTGCTCACTAAAGATGCGGGTAAGCTGACAATGGGCCAGCCCTTGGTGATATTGGCTCCGCATGCAGTCGAAGCATTGGTGAAGCAACCACCGGATCGGTGGCTTTCGAATGCGCGGATGACACACTACCAAGCACTTCTCCTGGATACAGACCGGGTGCAGTTTGGGCCTGTTGTTGCACTGAATCCTGCGACATTGTTGCCACTGCCGGAGGAAGGGCTCCAGCATAACTGCCTGGACATTCTGGCGGAAGCGCATGGGACGAGGCCAGATCTGACAGATCAACCACTGCCGGACGCAGATCATACATGGTATACAGACGGCTCGTCGTTGCTCCAAGAGGGTCAACGCAAGGCCGGCGCGGCTGTGACTACCGAGACTGAAGTCATCTGGGCGAAGGCCCTTCCCGCGGGCACCTCTGCCCAGCGCGCGGAACTTATTGCTCTGACACAAGCCTTGAAGATGGCCGAAGGCAAGAAGCTGAATGTCTATACGGACTCCCGGTATGCCTTTGCTACCGCTCACATACACGGAGAGATTTATAGGCGCCGCGGCTGGCTCACCTCTGAAGGAAAGGAGATAAAGAACAAAGATGAAATCCTCGCACTGCTGAAGGCGTTGTTCTTGCCCAAGCGGTTGTCGATTATACACTGTCCCGGACATCAGAAAGGACACTCTGCTGAGGCCCGCGGCAATAGGATGGCCGATCAGGCCGCGAGGAAAGCCGCGATCACGGAGACTCCGGACACTTCGACCTTGTTGATCGAGAATTCCTCTCCCTCCGGTGGCAGCAAACGGACTGCAGATGGTTCAGAGTTCGAACCAAAGAAGAAACGCAAGGTCTAACAATTCGGTACGCTGAAATCACCAGTCTCTCTCTACAAATCTATCTCTCTCTATTTTCTCCATAAATAATGTGTGAGTAGTTTCCCGATAAGGGAAATTAGGGTTCTTATAGGGTTTCGCTCATGTGTTGAGCATATAAGAAACCCTTAGTATGTATTTGTATTTGTAAAATACTTCTATCAATAAAATTTCTAATTCCTAAAACCAAAATCCAGTACTAAAATCCAGATCTCCTAAAGTCCCTATAGATCTTTGTCGTGAATATAAACCAGACACGAGACGACTAAACCTGGAGCCCAGACGCCGTTCGAAGCTAGAAGTACCGCTTAGGCAGGAGGCCGTTAGGGAAAAGATGCTAAGGCAGGGTTGGTTACGTTGACTCCCCCGTAGGTTTGGTTTAAATATGATGAAGTGGACGGAAGGAAGGAGGAAGACAAGGAAGGATAAGGTTGCAGGCCCTGTGCAAGGTAAGAAGATGGAAATTTGATAGAGGTACGCTACTATACTTATACTATACGCTAAGGGAATGCTTGTATTTATACCCTATACCCCCTAATAACCCCTTATCAATTTAAGAAATAATCCGCATAAGCCCCCGCTTAAAAATTGGTATCAGAGCCATGAATAGGTCTATGACCAAAACTCAAGAGGATAAAACCTCACCAAAATACGAAAGAGTTCTTAACTCTAAAGATAAAAGATCTTTCAAGATCAAA

B

SK-epegRNA vector:

*Bgl*II *Nhe*I *Sal*I

CGGCCGCAGATCTGCTAGCGTCGACGATTAAGCTTCGTTGAACAACGGAAACTCGACTTGCCTTCCGCACAATACATCATTTCTTCTTAGCTTTTTTTCTTCTTCTTCGTTCATACAGTTTTTTTTTGTTTATCAGCTTACATTTTCTTGAACCGTAGCTTTCGTTTTCTTCTTTTTAACTTTCCATTCGGAGTTTTTGTATCTTGTTTCATAGTTTGTCCCAGGATTAGAATGATTAGGCATCGAACCTTCAAGAATTTGATTGAATAAAACATCTTCATTCTTAAGATATGAAGATAATCTTCAAAAGGCCCCTGGGAATCTGAAAGAAGAGAAGCAGGCCCATTTATATGGGAAAGAACAATAGTATTTCTTATATAGGCCCATTTAAGTTGAAAACAATCTTCAAAAGTCCCACATCGCTTAGATAAGAAAACGAAGCTGAGTTTATATACAGCTAGAGTCGAAGTAGTGATTGGCTCGCAGGTGAACACAACACCTGCACACGTTTCAGAGC

*Aar*I *Aar*I

TATGCTGGAAACAGCATAGCAAGTTGAAATAAGGCTAGTCCGTTATCAACTTGA AAAAGTGGCACCGAGTCGGTGCAGAGACCATTGGTCTCTTCTCTCTC

*Bsa*I *Bsa*I Linker

CGCGGTTCTATCTAGTTACGCGTTAAACCAACTAGAATTTTTTTCCACATAAT

evopreQ_1_

CCTCGAGTCTAGAGGATCCTCGGTACC

*Xho*I *Xba*I *Bam*HI *Kpn*I

Target (*Aar*I):

5'-GGCANNNNNNNNNNNNNNNNNNN-3'

3'-NNNNNNNNNNNNNNNNNNNCAAA-5'

17-20 bp

pegRNA (*Bsa*I):

5'-GTGCNNNNNNNNNNNNNNNNNNN-3'

3'-NNNNNNNNNNNNNNNNNNNAAAA-5'

RT template PBS

C

SK-Nick gRNA vector:

*Bgl*II *Nhe*I *Sal*I

CGGCCGCAGATCTGCTAGCGTCGACGATTAAGCTTCGTTGAACAACGGAAACTCGACTTGCCTTCCGCACAATACATCATTTCTTCTTAGCTTTTTTTCTTCTTCTTCGTTCATACAGTTTTTTTTTGTTTATCAGCTTACATTTTCTTGAACCGTAGCTTTCGTTTTCTTCTTTTTAACTTTCCATTCGGAGTTTTTGTATCTTGTTTCATAGTTTGTCCCAGGATTAGAATGATTAGGCATCGAACCTTCAAGAATTTGATTGAATAAAACATCTTCATTCTTAAGATATGAAGATAATCTTCAAAAGGCCCCTGGGAATCTGAAAGAAGAGAAGCAGGCCCATTTATATGGGAAAGAACAATAGTATTTCTTATATAGGCCCATTTAAGTTGAAAACAATCTTCAAAAGTCCCACATCGCTTAGATAAGAAAACGAAGCTGAGTTTATATACAGCTAGAGTCGAAGTAGTGATTGGCTCGCAGGTGAACACAACACCTGCACACGTTTCAGAGC

*Aar*I *Aar*I

TATGCTGGAAACAGCATAGCAAGTTGAAATAAGGCTAGTCCGTTATCAACT

TGAAAAAGTGGCACCGAGTCGGTGCTTTTTTTCCACATAATCCTCGAGTCT

*Xho*I *Xba*I

AGAGGATCCTCGGTACC

*Bam*HI *Kpn*I

Nick sgRNA (*Aar*I):

5'-GGCANNNNNNNNNNNNNNNNNNN-3'

3'-NNNNNNNNNNNNNNNNNNNCAAA-5'

17-20 bp

**Fig. S1** **Complete sequences of the pC1300-PE, SK-epegRNA and SK-Nick gRNA vectors.** **A** The complete sequences of prime editor protein expression vector, pC1300-PE. The *2*x*35S* promoter is highlighted in blue upper-case characters, two NLS signal in grey upper-case characters, the nCas9 in purple upper-case characters, the linker in green upper-case characters, the engineered MMLV in yellow upper-case characters and the CaMV terminator in red upper-case characters. Restriction site *Kpn*I and *Bam*HI are indicated in red and yellow upper-case characters, respectively. **B** The complete sequences of epegRNAs expression vector, SK-epegRNA. The *AtU6* promoter is highlighted light green upper-case characters, and the gRNA scaffold is labeled in yellow. The two *Aar*I sites and two *Bsa*I sites are underlined. Target guide sequence can be inserted between two *Aar*I sites and pegRNAs can be inserted between two *Bsa*I sites. The RT template is highlighted in red upper-case characters and the evopreQ_1_ in brown upper-case characters. **C** The complete sequences of Nick gRNA expression vector, SK-Nick gRNA. The *AtU6* promoter is highlighted light green upper-case characters, and the gRNA scaffold is labeled in yellow. The two *Aar*I sites are underlined.


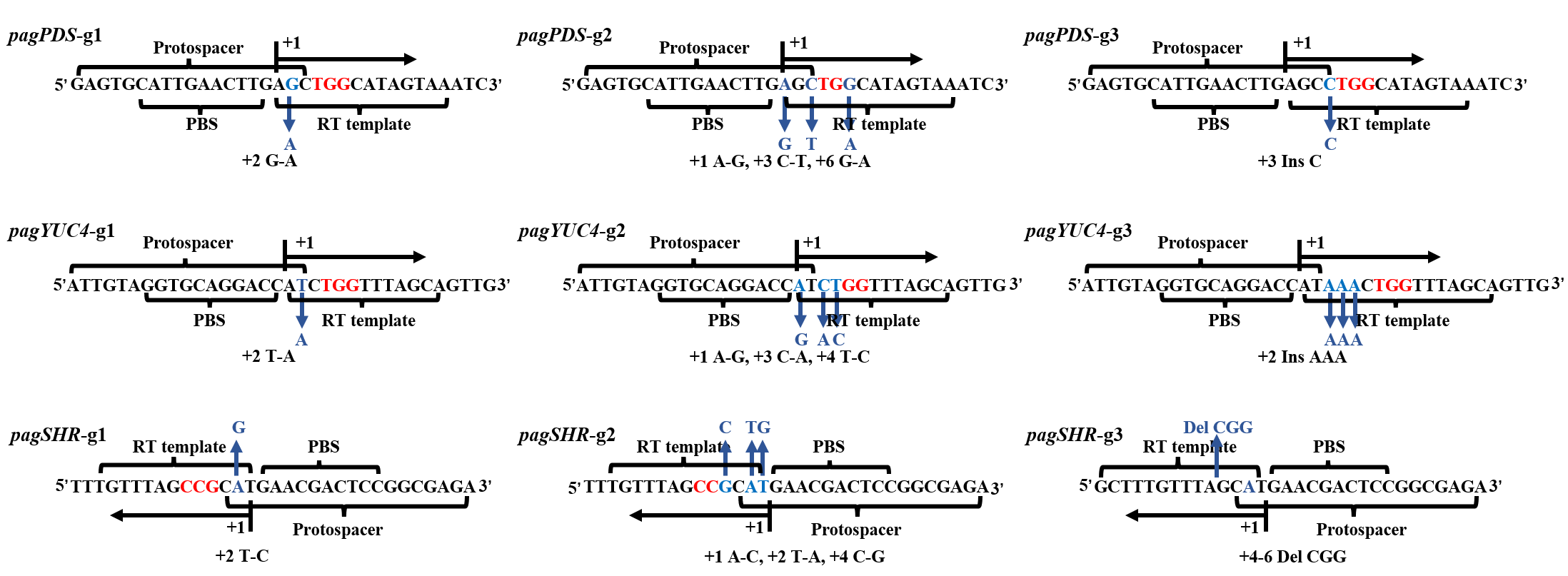


**Fig. S2** Schematic illustrations of pegRNAs design. The PAM used for epegRNA design is labeled in red. The desired edits are shown in blue. The position of the desired edits was counted from the pegRNA-nick site following the arrow.


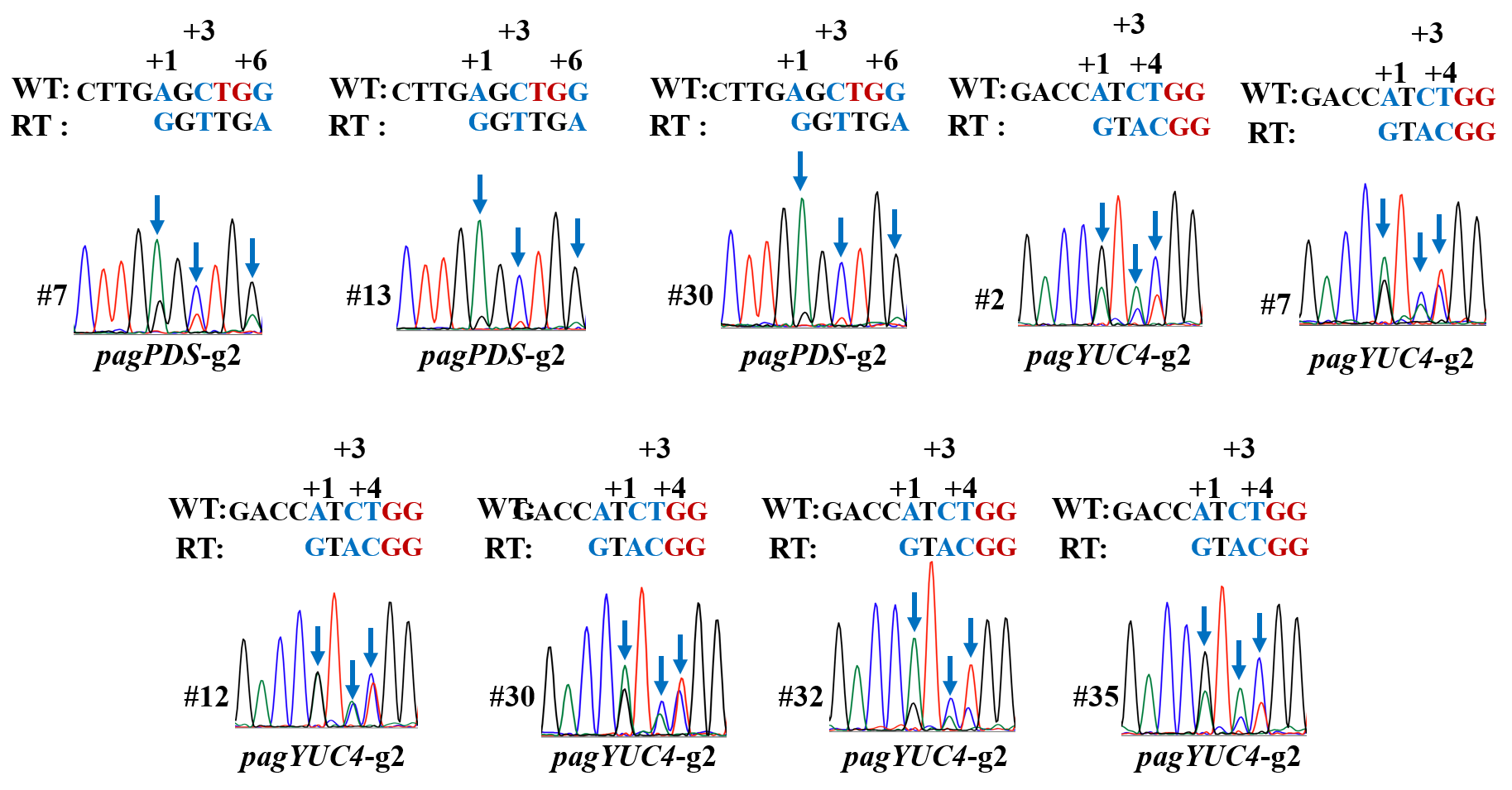


**Fig. S3** Sanger sequencing of other desired edits in T_0_ plants. The desired edits are shown in blue. The blue arrows indicate the location of desired edits. The PAM used is labeled in red.


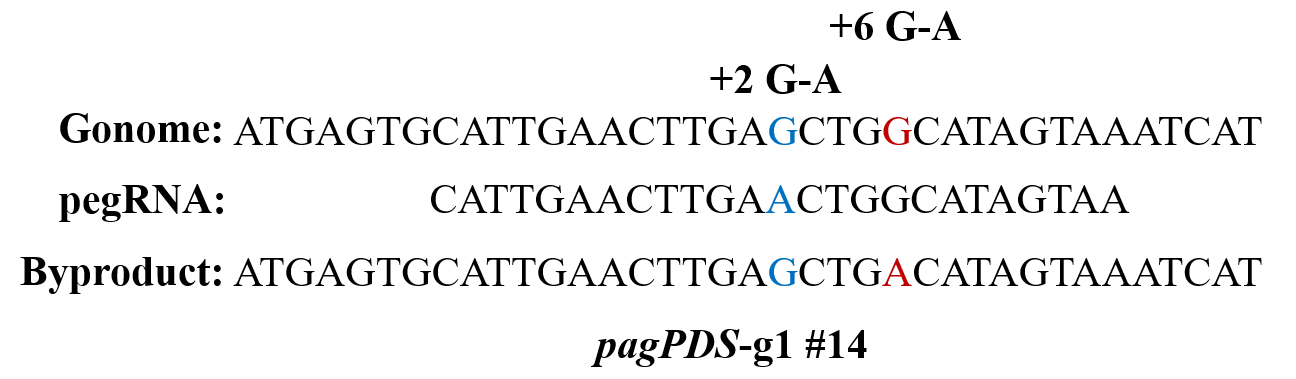


**Fig. S4** Alignments of PE byproducts at *pagPDS-g1* site in T_0_ plants. The desired edits are shown in blue. The byproducts are shown in red.

**Supplementary Table 1 pegRNA target sites, Nick sgRNA, RT template and PBS of different target sites.**

| Genes | Target sequence | Sequence for RT template and PBS | nicking sgRNA | nicking position | Desired edit |
| --- | --- | --- | --- | --- | --- |
| *pagPDS*-g1 | GAGTGCATTGAACTTGAGC**TGG** | TTACTATGCCAGTTCAAGTTCAATG | TAGCAGAAGAATTTCCAAAT**GGG** | nick+131 | +2 G-A |
| *pagYUC4*-g1 | ATTGTAGGTGCAGGACCATC**TGG** | TGCTAAACCAGTTGGTCCTGCAC | GGGTACTTGGGGAAATCATC**AGG** | nick+166 | +2 T-A |
| *pagSHR*-g1 | TCTCGCCGGAGTCGTTCATG**CGG** | TGTTTAGCCGCGTGAACGACTCC | AATGAGCTTGGTTCACCTTA**TGG** | nick+60 | +2 T-C |
| *pagPDS*-g2 | GAGTGCATTGAACTTGAGC**TGG** | GATTTACTATGTCAACCCAAGTTCAATG | TAGCAGAAGAATTTCCAAAT**GGG** | nick+131 | +1 A-G, +3 C-T, +6 G-A |
| *pagYUC4*-g2 | ATTGTAGGTGCAGGACCATC**TGG** | ACTGCTAAACCGTACGGTCCTGCAC | GGGTACTTGGGGAAATCATC**AGG** | nick+166 | +1 A-G, +3 C-A, +4 T-C |
| *pagSHR*-g2 | TCTCGCCGGAGTCGTTCATG**CGG** | TTGTTTAGCCCCTGGAACGACTCC | AATGAGCTTGGTTCACCTTA**TGG** | nick+60 | +1 A-C, +2 T-A, +4 C-G |
| *pagPDS*-g3 | GAGTGCATTGAACTTGAGC**TGG** | TTTACTATGCCAGGCTCAAGTTCAATG | TAGCAGAAGAATTTCCAAAT**GGG** | nick+131 | +3 Ins C |
| *pagYUC4*-g3 | ATTGTAGGTGCAGGACCATC**TGG** | TGCAAAACCAGTTTATGGTCCTGCAC | GGGTACTTGGGGAAATCATC**AGG** | nick+166 | +2 Ins AAA |
| *pagSHR*-g3 | TCTCGCCGGAGTCGTTCATG**CGG** | GCTTTGTTTAGCATGAACGACTCC | AATGAGCTTGGTTCACCTTA**TGG** | nick+60 | +4-6 Del CGG |

The PAM motif in each target sequence and nicking sgRNA are shown in bold. The PBS sequence are underlined. The RT template are shown in red.

**Supplementary Table 2 NGS primers were used to identify the precise editing types of the T_0_ plants.**

| Targets | Primer name | Primer Sequence (5'-3') |
| --- | --- | --- |
| *pagPDS* | *pagPDS*-HF | ggagtgagtacggtgtgcCAGGGCTGTTGTTACAGTTG |
|  | *pagPDS*-HR | gagttggatgctggatggTGAGAACAAGTAGGATAAGC |
| *pagYUC4* | *pagYUC4*-HF | ggagtgagtacggtgtgcAAGCAAAGTTCGTTAAGGTT |
|  | *pagYUC4*-HR | gagttggatgctggatggAGAGAGAAGCTATGCAGTCA |
| *pagSHR* | *pagSHR*-HF | ggagtgagtacggtgtgcACACAGAGCAAAAACTTGCT |
|  | *pagSHR*-HR | gagttggatgctggatggTAGTCCAAGGACTCACCTCT |

The red and green sequences represent barcode primers.

**Supplementary Table 3 PCR primers for amplifying the target sites and Sanger sequence of T_0_ plants.**

| Targets | Primer name | Primer Sequence (5'-3') |
| --- | --- | --- |
| *pagPDS* | *pagPDS*-SF | GACTTCCTTGCTTCAATCTTG |
|  | *pagPDS*-SR | ACCGTGTTATCAAGGTCCGG |
| *pagYUC4* | *pagYUC4*-SF | TGGATTCTTGTAAAGAGCAA |
|  | *pagYUC4*-SR | GGCTTGGTTGAATTTAGGCT |
| *pagSHR* | *pagSHR*-SF | CCACTACTACCACCAACACT |
|  | *pagSHR*-SR | TGGTTAACTTCAAGTGTGGT |
